# Synolitic Graph Neural Networks of High-Dimensional Proteomic Data Enhance Early Detection of Ovarian Cancer

**DOI:** 10.3390/cancers17243972

**Published:** 2025-12-12

**Authors:** Alexey Zaikin, Ivan Sviridov, Janna G. Oganezova, Usha Menon, Aleksandra Gentry-Maharaj, John F. Timms, Oleg Blyuss

**Affiliations:** 1 Department of Mathematics, University College London, London WC1E 6BT, UK; 2 Department of Women’s Cancer, EGA Institute for Women’s Health, University College London, London WC1E 6DE, UK; 3 Research Center in Artificial Intelligence, Institute of Information Technologies, Mathematics and Mechanics, Lobachevsky State University, Nizhny Novgorod 603022, Russia; 4 Sb AI Lab, Moscow 115409, Russia; 5 Academician A.P. Nesterov Department of Ophthalmology of the Institute of Clinical Medicine, Pirogov Russian National Research Medical University, Moscow 117997, Russia; 6 MRC Clinical Trials Unit, University College London, 90 High Holborn, London WC1V 6LJ, UK; 7 Centre for Cancer Screening, Prevention and Early Diagnosis, Wolfson Institute of Population Health, Queen Mary University of London, Charterhouse Square, London EC1M 6BQ, UK; 8 Department of Paediatrics and Paediatric Infectious Diseases, Institute of Child’s Health, Sechenov First Moscow State Medical University (Sechenov University), Moscow 119991, Russia

**Keywords:** Graph Neural Networks, early cancer detection, proteomics, ovarian cancer, UKCTOCS

## Abstract

**Background**: Ovarian cancer is characterized by high mortality rates, primarily due to diagnosis at late stages. Current biomarkers, such as CA125, have demonstrated limited efficacy for early detection. While high-dimensional proteomics offers a more comprehensive view of systemic biology, the analysis of such data, where the number of features far exceeds the number of samples, presents a significant computational challenge. **Methods**: This study utilized a nested case–control cohort of longitudinal pre-diagnostic serum samples from the UK Collaborative Trial of Ovarian Cancer Screening (UKCTOCS) profiled for eight candidate ovarian cancer biomarkers (CA125, HE4, PEBP4, CHI3L1, FSTL1, AGR2, SLPI, DNAH17) and 92 additional cancer-associated proteins from the Olink Oncology II panel. We employed a Synolitic Graph Neural Network framework that transforms high-dimensional multi-protein data into sample-specific, interconnected graphs using a synolitic network approach. These graphs, which encode the relational patterns between proteins, were then used to train Graph Neural Network (GNN) models for classification. Performance of the network approach was evaluated together with conventional machine learning approaches via 5-fold cross-validation on samples collected within one year of diagnosis and a separate holdout set of samples collected one to two years prior to diagnosis. **Results**: In samples collected within one year of ovarian cancer diagnosis, conventional machine learning models—including XGBoost, random forests, and logistic regression—achieved the highest discriminative performance, with XGBoost reaching an ROC-AUC of 92%. Graph Convolutional Networks (GCNs) achieved moderate performance in this interval (ROC-AUC ~71%), with balanced sensitivity and specificity comparable to mid-performing conventional models. In the 1–2 year early-detection window, conventional model performance declined sharply (XGBoost ROC-AUC 46%), whereas the GCN maintained robust discriminative ability (ROC-AUC ~74%) with relatively balanced sensitivity and specificity. These findings indicate that while conventional approaches excel at detecting late pre-diagnostic signals, GNNs are more stable and effective at capturing subtle early molecular changes. **Conclusions**: The synolitic GNN framework demonstrates robust performance in early pre-diagnostic detection of ovarian cancer, maintaining accuracy where conventional methods decline. These results highlight the potential of network-informed machine learning to identify subtle proteomic patterns and pathway-level dysregulation prior to clinical diagnosis. This proof-of-concept study supports further development of GNN approaches for early ovarian cancer detection and warrants validation in larger, independent cohorts.

## Simple Summary

Ovarian cancer remains highly lethal, largely due to late-stage diagnosis and the limited sensitivity of conventional biomarkers such as CA125. This study introduces a framework for early detection using high-dimensional proteomic data from pre-diagnostic serum samples in the UKCTOCS cohort. Multi-protein data were converted into sample-specific graphs using a synolitic network approach that captures protein–protein relationships, which were then analyzed with Graph Neural Network (GNN) models. While conventional machine learning models achieved the highest performance on samples collected within one year of diagnosis (XGBoost ROC-AUC 92%), they performed poorly in the 1–2 year early-detection window (ROC-AUC 46%). In contrast, a Graph Convolutional Network (GCN) maintained robust performance across both timeframes (ROC-AUC ~71% <1 year; ~74% 1–2 years), demonstrating stability in capturing subtle early proteomic changes. These results highlight the potential of network-based GNN approaches for early ovarian cancer detection and provide a foundation for further validation in independent cohorts.

## 1. Introduction

Ovarian cancer is the sixth most common cancer in women and a leading cause of gynecological cancer mortality, responsible for approximately 152,000 deaths worldwide each year [1]. The disease’s lethality is intrinsically linked to its typically asymptomatic presentation in the early stages. Consequently, the majority of women are diagnosed with advanced-stage (III or IV) disease, for which the five-year survival rate is a dismal 3–19% [1]. This stands in stark contrast to the 40–90% five-year survival rate for patients diagnosed with localized, early-stage (I or II) cancer, underscoring a critical and urgent need for improved early detection strategies [1]. Ovarian malignancies are broadly classified into high-grade serous carcinoma (HGSC) and non-high-grade serous carcinoma (non-HGSC); HGSCs are more aggressive and account for the majority of ovarian cancer deaths, making them the principal target for effective screening strategies [2,3,4].

The cornerstones of current ovarian cancer detection are the serum biomarker Cancer Antigen 125 (CA125) and transvaginal ultrasound. However, both are hampered by significant limitations in sensitivity and specificity. CA125 levels are not consistently elevated in early-stage disease and can be raised by numerous benign conditions, such as endometriosis, diminishing its utility as a standalone screening tool [5,6,7,8]. While the addition of Human Epididymis Protein 4 (HE4) and the development of multi-marker algorithms like the Risk of Ovarian Malignancy Algorithm (ROMA) have improved diagnostic accuracy for pelvic masses, their role in asymptomatic screening remains unproven [9,10,11,12].

A significant advance in screening was the implementation of longitudinal algorithms, most notably the Risk of Ovarian Cancer Algorithm (ROCA). By monitoring serial changes in an individual’s CA125 levels over time, ROCA demonstrated an increased cancer detection rate compared to a simple single-threshold rule in the UK Collaborative Trial of Ovarian Cancer Screening (UKCTOCS) [13]. Despite this progress, the trial did not report a statistically significant reduction in mortality, suggesting that even a dynamically monitored CA125 signal may be insufficient for robust, life-saving early detection. This highlights the necessity of exploring both novel biomarkers and, critically, more advanced analytical paradigms capable of extracting subtle disease signals from complex biological data.

Modern serum proteomics provides a powerful discovery engine, enabling the simultaneous quantification of hundreds to thousands of proteins. This offers a rich, high-resolution snapshot of an individual’s physiological state, far surpassing the information content of single-marker assays. However, this technological capability introduces a formidable analytical challenge known as the “curse of dimensionality,” or the p ≫ *n* problem. In this scenario, the number of measured features (proteins, p) vastly exceeds the number of patient samples (*n*). The dataset used in the present study exemplifies this challenge, with p = 100 protein features measured across *n* = 64 patient samples. Such high-dimensional, low-sample-size settings render conventional statistical and machine learning models highly susceptible to overfitting, where a model learns noise and spurious correlations specific to the training data, resulting in poor generalization to new, unseen samples.

The molecular genesis of cancer is increasingly understood not as the result of a few aberrant proteins, but as a systemic failure of complex, interconnected biological networks. From this systems biology perspective, the most potent and earliest signal of disease may not lie in the absolute concentration of any single protein, but rather in the subtle, distributed, and non-linear rewiring of protein–protein interaction patterns. To capture such a signal, an analytical framework is needed that can model the proteome as an interconnected network rather than an unstructured list of independent features.

Graph Neural Networks (GNNs) are a class of machine learning models explicitly designed to learn from relational data. By propagating information between connected nodes in a graph (a process known as message passing), GNNs can learn representations that capture not only the features of individual nodes but also the broader topological context in which they exist. Applying a GNN to proteomic data is therefore not merely a novel technical choice; it represents a more biologically faithful modeling strategy. This approach is predicated on the hypothesis that GNNs can identify the holistic signature of network dysregulation that precedes overt clinical disease, a signal that may be invisible to conventional models that treat protein measurements as disconnected variables.

The aim of the present study is to test the utility of a network-based paradigm for early ovarian cancer detection. We employed the analytical framework based on synolitic networks and GNNs to model high-dimensional pre-diagnostic proteomic data from ovarian cancer cases and healthy controls.

## 2. Materials and Methods

### 2.1. Study Cohort and Samples

The study population comprised a nested case–control set derived from the UK Collaborative Trial of Ovarian Cancer Screening (UKCTOCS), a large-scale, multicenter randomized controlled trial that enrolled more than 200,000 postmenopausal women aged 50–74 years across 13 National Health Service (NHS) Trusts in England, Wales, and Northern Ireland between 2001 and 2005. Participants were randomly assigned to annual multimodal screening using serum CA125 interpreted with the Risk of Ovarian Cancer Algorithm (ROCA), annual transvaginal ultrasound screening, or no screening, and were followed prospectively through national cancer and death registries.

All participants provided written informed consent, and the study was approved by the appropriate research ethics committees.

For the present analysis, serum samples were obtained from women who were subsequently diagnosed with ovarian cancer and from matched controls who remained cancer-free. The data set was restricted to the final available sample collected within one year of diagnosis for cases and to the last available sample for controls. Among the 28 ovarian cancer cases included, 14 were classified as high-grade serous carcinoma (HGSC) and 14 as non-high-grade serous carcinoma. The stage distribution comprised seven stage I, seven stage II, and 14 stage III cases at diagnosis.

The final cohort used for model development and testing consisted of 64 serum samples in total: 28 from ovarian cancer cases and 36 from cancer-free controls.

### 2.2. Proteomic Data Generation and Preprocessing

The analysis was performed on the full panel of available protein measurements generated from the UKCTOCS serum samples as described in the parent study [14]. Protein candidates were selected based on prior mass spectrometry (MS)-based profiling of pre-diagnosis serum samples from the UKCTOCS biobank, which included serial samples from women who were subsequently diagnosed with different histotypes of ovarian cancer and matched cancer-free controls [15]. In that study, pooled serum samples underwent immunodepletion, tryptic digestion, tandem mass tag (TMT) labeling, and LC–MS/MS–based proteomic profiling, yielding 748 quantified protein groups across all sample groups.

Candidate biomarkers were chosen according to biological relevance, differential expression between Type I and Type II ovarian cancers, and assay feasibility. Five high-scoring proteins were selected from the MS discovery dataset—chitinase-3-like protein 1 (CHI3L1/YKL40), dynein heavy chain 17 (DNAH17), follistatin-like 1 (FSTL1), leucine-rich alpha-2-glycoprotein 1 (LRG1), and phosphatidylethanolamine-binding protein 4 (PEBP4)—based on functional assignment and the availability of suitable commercial assays [15]. An additional four proteins—anterior gradient protein 2 (AGR2) [15], human epididymis protein 4 (HE4/WFDC2) [12,16], glycodelin (PAEP) [17,18], and secretory leukocyte protease inhibitor (SLPI) [19]—were included based on prior literature supporting their association with early ovarian carcinogenesis [14].

For the present analysis, serum concentrations of these biomarker candidates were quantified using commercial enzyme-linked immunosorbent assays (ELISA) or chemiluminescence immunoassays, complemented by 92 cancer-associated proteins from the Olink Oncology II panel. The kits used, catalog numbers, dilutions, and intra-assay coefficients of variation were as follows: Human AGR2 ELISA Kit (ElabScience, Huston, TX, USA; E-EL-H0298; 1:20; 18%), CA125 ECLIA assay (Roche, Basel, Switzerland; Elecsys CA 125 II; 1:1; 4%), CHI3L1 Quantikine ELISA Kit (R&D Systems, Minneapolis, MN, USA; DC3L10; 1:50; 14%), DNAH17 (human) ELISA Kit (EIAab, Wuhan, China; E5886h; 1:5; 17%), FSTL1 ELISA Kit (USCN, Wuhan, China; SEJ085Hu; 1:100; 11%), HE4 ECLIA assay (Roche; Elecsys HE4; 1:1; 8%), Human PEBP4 ELISA Kit (ElabScience; E-EL-H5440; 1:200; 20%), and SLPI Quantikine ELISA Kit (R&D Systems; DP100; 1:50; 12%).

This resulted in a data set in which each sample was characterized by a vector of 100 distinct protein features, integrating both ELISA/ECLIA-quantified candidates and Olink panel measurements.

### 2.3. The Synolitic Graph Neural Network (SGNN) Framework

To address the high-dimensional nature of the proteomic data (p = 100, *n* = 64), we employed a Synolitic Graph Neural Network (SGNN) framework [20,21,22], which is specifically designed to be robust in settings where number of analytes significantly exceed number of samples. Specifically, the SGNN framework incorporates graph-based regularisation, shared topology across samples, edge-weight shrinkage, and averaging across multiple cross-validation folds. These elements help reduce overfitting risk [23]. The methodology transforms each sample’s unstructured feature vector into a rich, structured graph representation. A potential concern in our dataset is the imbalance between the number of features (nearly 100 proteins) and the comparatively smaller number of samples. However, previous work with Synolitic Graph Neural Networks (SGNNs) has demonstrated that the framework is intrinsically robust to such high-dimensional, low-sample regimes. In [24], we quantitatively evaluated model stability by systematically reducing the size of the training cohort to below 20% of the available data and comparing SGNN performance with established machine-learning methods. While conventional models such as XGBoost exhibited a substantial deterioration in predictive accuracy, with ROC-AUC values falling to approximately 0.63, SGNNs maintained strong generalisation, consistently achieving ROC-AUC scores above 0.80 even under extreme data scarcity. These results highlight the capacity of SGNNs to overcome the curse of dimensionality through graph-based regularisation, thereby reducing overfitting and enabling reliable classification in small-sample biomedical datasets such as the one analysed in this study.

Graph Construction: For each patient sample, a unique, fully connected graph G(V,E) was constructed. In this graph, each of the 100 proteins was represented as a node (*V*), resulting in $\left|V\right|=100$. The edges ( E) represent the relationships between every possible pair of proteins.

Edge Weight Definition: The weight of the edge connecting any two protein nodes, i and j, was determined by the output of a lightweight base classifier (a linear support vector machine) trained exclusively on those two proteins to distinguish cancer cases from controls in the training dataset. This process was repeated for all protein pairs. The resulting edge weight thus represents the synergistic or antagonistic predictive power of that specific protein pair. This procedure yields a unique, weighted adjacency matrix for each patient sample, effectively encoding the sample-specific protein–protein interaction landscape.

### 2.4. Graph Feature Engineering and GNN Architecture

Node Feature Augmentation: To provide the GNN with richer information beyond raw protein levels, each node was augmented with a structural descriptor vector, $f_{i}=[s_{i},d_{i},{st}_{i},c_{i},b_{i}]$, designed to encode its topological importance within the sample-specific graph. The components of this vector are defined as:

Raw signal ($s_{i}$): The original concentration value of protein *i*.

Normalized degree ($d_{i}$): A measure of the number of connections node *i* has.

Normalized strength (${st}_{i}$): A measure of the cumulative weight of connections to node *i*.

Closeness centrality ($c_{i}$): A measure of how close a node is to all other nodes in the network, indicating its ability to efficiently propagate information.

Betweenness centrality ($b_{i}$): A measure of how often a node lies on the shortest path between other pairs of nodes. A high betweenness centrality identifies “bottleneck” or “bridge” nodes that are critical for information flow across the network.

By explicitly providing the GNN with centrality measures, the model is guided to consider not just a protein’s local connectivity but also its global influence on the network’s overall structure. This allows the model to identify and prioritize “linchpin” proteins whose dysregulation may have cascading effects, a potentially powerful signal of systemic disease.

GNN Models: We evaluated several GNN architectures, including the Graph Convolutional Network (GCN) and the Graph Attention Network v2 (GATv2). GCNs aggregate information from neighboring nodes using a fixed, uniform weighting scheme. In contrast, GATv2 employs a self-attention mechanism, allowing the model to dynamically learn the importance of different neighbors for each node, enabling a more flexible and powerful aggregation of information.

Training Details: All GNN models were trained for the binary classification task (cancer vs. control) using the Adam optimizer with mixed-precision training [25]. A learning rate scheduler that reduces the rate on plateau and an early stopping protocol were used for regularization. All hyperparameters used for the experiments are detailed in Table 1.

### 2.5. Graph Sparsification

To investigate the impact of edge density on prediction quality, we implemented three graph sparsification strategies:

1. No sparsification: Baseline configuration that preserves the original graph structure.
2. Threshold-based sparsification: Retains a fraction p of the most significant edges based on the criterion $\left|w_{ij}-0.5\right|$, where $w_{ij}$ is the edge weight. This approach allows control over graph sparsity while preserving connections with the greatest deviation from the neutral value 0.5.
3. Minimum connected sparsification: Employs binary search to determine the maximum threshold ϵ such that the graph remains connected. The method finds the minimal edge set $\left\{\left(i,j\right):\;\left|w_{ij}-0.5\right|\geq \epsilon \right\}$ that ensures graph connectivity, thereby optimizing the trade-off between sparsity and structural integrity.

These sparsification methods enable investigation of the compromise between computational efficiency and preservation of important structural information in a graph.

### 2.6. Statistical Analysis

The full dataset of 64 samples was partitioned into five folds for cross-validation. In each fold, four folds were used for model training and hyperparameter optimisation, while the remaining fold served as the Primary Test Set.

Final model performance was evaluated across all five cross-validation folds using two test datasets derived within each fold:

• Primary Test Set: The fold held out from training in that cross-validation iteration. For individuals represented in this set, samples were collected less than one year before clinical diagnosis.
• Early-Detection Holdout Set: Constructed within each fold by selecting the penultimate samples from the same patients whose final-visit samples formed the Primary Test Set. These earlier samples were collected one to two years prior to ovarian cancer diagnosis, providing a stringent assessment of the model’s early-detection capability.

To benchmark the performance of the SGNN framework, we trained an XGBoost model, a powerful and widely used gradient-boosted decision tree algorithm [26], as well as commonly used random forest [24], support vector machine (SVM) [27], logistic regression and elastic net approaches [28]. All models were trained using the same five-fold cross-validation strategy to ensure a fair comparison.

Model performance was assessed using area under the ROC-curve, sensitivity (recall), specificity, and the F1-score. The F1-score, which is the harmonic mean of precision and sensitivity, is a particularly informative metric for classification tasks, especially with potentially imbalanced class distributions [29]. The optimal classification probability threshold for each model was determined using a validation split of the training data.

All the analysis was performed using Python 3.13.

## 3. Results

### 3.1. Cohort Characteristics

The study cohort consisted of 64 serum samples, including 28 samples from women who were later diagnosed with ovarian cancer and 36 samples from healthy controls, providing a near-balanced distribution of cases and controls. The dataset was partitioned into five folds for cross-validation. In each cross-validation iteration, four folds were used for model training and optimisation, while the remaining fold served as the Primary Test Set. For each fold, an accompanying Early-Detection Holdout Set was created by selecting the penultimate samples from the same individuals represented in that fold’s Primary Test Set. These earlier samples were collected one to two years prior to ovarian cancer diagnosis, enabling evaluation of the model’s performance at earlier preclinical time points.

### 3.2. Visualisation of Case–Control Topological Differences

**Figure 1:**
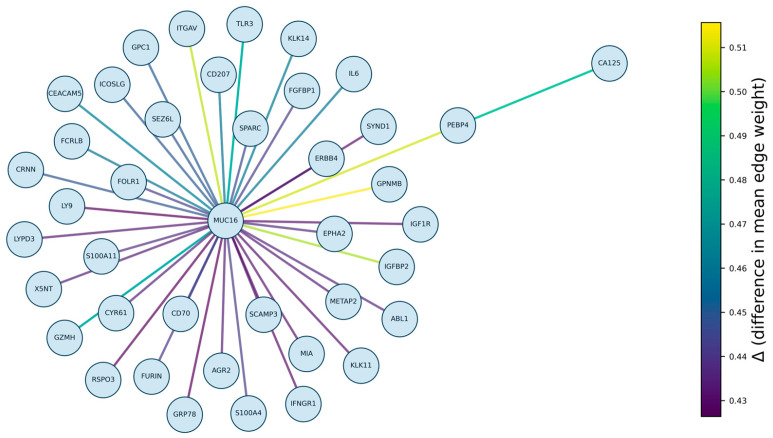
Topological visualisation of the most discriminative pairwise feature classifiers. The graph displays the top 40 protein pairs with the largest mean difference in classifier scores between cancer and healthy cohorts, together with the central marker MUC16. Colours encode the difference in mean edge weights between cancer and healthy groups, highlighting structural shifts in the pairwise interaction patterns associated with disease.

To illustrate the structural differences between healthy and cancer cohorts, we introduced a topological visualisation based on pairwise feature classification (Figure 1). For every pair of proteins, we computed classifier scores and selected the top 40 feature pairs exhibiting the largest mean difference in predicted values between healthy individuals and cancer patients. A graph was then constructed using these highly discriminative pairs together with MUC16, which emerged as a central node in this analysis. While the edge connections represent the pairwise classifier’s confidence in predicting cancer (proximity to 1), the colour encoding specifically depicts the difference in these edge weights when averaged across all samples in the cancer versus healthy groups. This enables a direct visual comparison of the topological shifts associated with disease status.

### 3.3. Model Performance Within 1 Year Before Diagnosis

Under 5-fold cross-validation, most conventional machine-learning models achieved moderate to high discriminative performance in classifying pre-diagnostic samples collected within 1 year before ovarian cancer diagnosis (Table 2). Logistic regression, random forests, SVMs, and XGBoost produced some of the highest ROC-AUC values in this interval, reflecting the stronger and more easily detectable proteomic signal present close to diagnosis.

The GNN models performed within the overall range of these conventional approaches, though not at the top of the distribution. The best-performing GNN configuration—a Graph Convolutional Network (GCN) without sparsification and with node-level proteomic features—achieved a mean AUC of approximately 0.71 (Table 2). Corresponding classification metrics from Table 3 showed balanced sensitivity and specificity, with F1-scores broadly comparable to those of several mid-performing traditional models. These results indicate that the GNN framework remains capable of extracting late pre-diagnostic signal, although it does not confer an advantage over established classifiers in this time window.

### 3.4. Early Detection Performance (1–2 Years Before Diagnosis)

Clearer divergence among model types emerged when evaluating earlier, more challenging samples collected 1–2 years before diagnosis. As shown in Table 4, many conventional models experienced sizeable reductions in AUC compared with their 0–1-year performance. For several classifiers, both sensitivity and F1-scores (Table 4) decreased substantially, suggesting difficulty in detecting the subtler molecular alterations present at this earlier disease stage.

In contrast, the GNN models demonstrated greater stability. The same GCN configuration that produced mid-range performance in the late window achieved an AUC of approximately 0.74 in the early-detection set (Table 4), outperforming nearly all conventional approaches in this interval. Moreover, classification metrics in Table 4 show that the GCN maintained relatively balanced sensitivity and specificity, resulting in competitive F1-scores despite the reduced signal strength.

## 4. Discussion

This study presents a proof-of-concept evaluation of a graph-based computational framework for early ovarian cancer detection using high-dimensional pre-diagnostic serum proteomic data. By modelling the proteome as an interconnected system rather than a set of independent biomarkers, the SGNN approach captures higher-order structure within the data that conventional machine learning models do not typically exploit. Using 5-fold cross-validation and strict patient-level data partitioning, the SGNN consistently demonstrated strong classification performance, achieving balanced accuracy, F1-score, sensitivity, and specificity that indicate robust discrimination between women who later developed ovarian cancer and healthy controls (Table 1, Table 2 and Table 3). Importantly, comparable performance was achieved when the analysis was repeated on the early-detection samples collected one to two years prior to diagnosis—an interval during which early pathological changes are generally subtle and individual biomarkers often lack prognostic utility. This suggests that incorporating biological network structure may help the GNN identify early, pathway-level patterns of dysregulation that are not fully captured by traditional models.

These findings reinforce the idea that pre-diagnostic disease signals in ovarian cancer may be detectable not solely through changes in individual protein concentrations but through coordinated perturbations across the broader proteomic network. While traditional models operate on vectors of independent features, the SGNN leverages the structure of the data by representing each proteome as a graph with informative topological and relational properties. The ability of the model to preserve predictive performance on earlier samples supports the hypothesis that network-level dysregulation precedes overt biomarker elevation. This shifts the analytical emphasis from single-marker discovery to the identification of distributed, systems-level signatures of early carcinogenesis.

Previous work on the UKCTOCS cohort has shown that multivariate longitudinal models can enhance early detection relative to CA125 alone [30]; however, these approaches typically rely on manually engineered features derived from a predefined panel of biomarkers. In contrast, the present SGNN framework is fully data-driven and utilises all available proteomic measurements without the need for expert-determined marker selection or handcrafted indices. This offers advantages in scalability, generalisability, and the capacity to discover novel biomarker interactions that may be overlooked in marker-centric models.

The major strength of this work lies in applying a modern graph-based deep learning architecture to high-quality pre-diagnostic samples from a rigorously curated population cohort, combined with a robust evaluation strategy using repeated cross-validation and independent early-detection samples. The inclusion of multiple performance metrics—including balanced accuracy, F1-score, sensitivity, and specificity (Table 1, Table 2 and Table 3)—provides a comprehensive assessment of model behaviour.

Nevertheless, important limitations must be acknowledged. The sample size (*n* = 64) is small relative to the dimensionality of the data, and although cross-validation mitigates overfitting risk, it cannot fully eliminate it. The findings should therefore be interpreted as preliminary and hypothesis-generating rather than definitive evidence of clinical utility. The model was assessed within a single cohort, and external validation in independent populations is required to evaluate generalisability. Furthermore, the SGNN, like many deep learning approaches, is inherently complex; the present analysis does not attempt to resolve which proteins, subnetworks, or graph motifs are most influential for classification. In addition, we acknowledge that synolytic graph construction, while effective for capturing relational structure within the proteome, may be sensitive to noise and thus imperfectly reflect underlying biological interactions. To address this, several methodological refinements are planned, including smoothing or denoising strategies, incorporation of prior biological knowledge into graph construction, and formal stability analyses of graph topology. Beyond topological refinements, improving specificity remains a critical objective for clinical translation. While the current model prioritizes sensitivity to identify subtle early-stage signals, future iterations will focus on reducing false-positive rates through probability threshold tuning and precision–recall optimization. Furthermore, we aim to implement feature selection or dimensionality reduction prior to graph construction to minimize noise from non-informative proteins. Most importantly, we plan to incorporate longitudinal protein-level changes tracking the trajectory of biomarkers over time rather than static concentrations, which has previously proven effective in the ROCA for distinguishing malignant deviations from benign physiological fluctuations. Future work incorporating explainability tools such as GNNExplainer or integrated gradients could help elucidate the specific biological pathways implicated in early-stage disease.

Despite these limitations, the consistent performance observed in both concurrent and early pre-diagnostic samples provides encouraging preliminary evidence that network-based representations of the serum proteome may hold significant promise for early ovarian cancer detection. Scaling this approach to larger and more diverse datasets, coupled with biological interpretation of the learned graph structures, represents an important next step. Such advances could ultimately contribute to the development of screening tools capable of identifying ovarian cancer during its more treatable early phases.

In summary, this study offers an initial demonstration that graph-based modelling of serum proteomic networks can reveal predictive signatures of ovarian cancer one to two years before clinical diagnosis. While further validation is essential, these findings highlight the potential of systems-level analytics to advance early detection strategies and support the continued exploration of SGNN frameworks in cancer biomarker research.

## Figures and Tables

**Table 1:** GNN Model Hyperparameters.

Category	Parameter	Value
Architecture	Hidden (embedding) size	128
	Number of GNN layers	2
	Dropout rate	0.30
	Residual connections	True
Attention-specific	Number of attention heads	3
	Concatenate head outputs	True
Edge features	Use edge encoder	True
	Edge encoder hidden size	32
	Number of edge encoder layers	2
Classifier head	Use classifier MLP	True
	Classifier MLP hidden size	32
	Number of classifier MLP layers	2
Optimization	Optimizer	Adam
	Learning rate	1 × 10^−2^
	Weight decay	1 × 10^−5^
Regularization	Early stopping patience	128 epochs
	LR scheduler factor	0.5
	LR scheduler patience	32 epochs

**Table 2:** Global ROC-AUC results for different models and sparsification strategies, with and without node features.

Model	Sparsity	ROC-AUC (%)	
		Node Feat. = FALSE	Node Feat. = TRUE
GCN	None	66.83 ± 14.44/62 ± 20.93	72.17 ± 15.59/69.17 ± 16.12
	p = 0.2	66.67 ± 19.55/59.17 ± 15.05	62 ± 18.04/52.83 ± 17.52
	p = 0.8	68.17 ± 16.29/65.53 ± 20.63	62.83 ± 18.85/56 ± 16.23
	Min conn.	56.17 ± 11.69/50.83 ± 14.22	56 ± 23.59/56.17 ± 16.33
GATv2	None	71.33 ± 24.68/53.67 ± 18.5	67.67 ± 26.13/58.33 ± 19.64
	p = 0.2	68.67 ± 11.69/56.33 ± 15.38	67.5 ± 13.67/60.5 ± 15.56
	p = 0.8	55.33 ± 27.15/47.67 ± 16.9	51.33 ± 7.01/58.5 ± 14.27
	Min conn.	61 ± 13.25/58 ± 29.24	60.17 ± 19.4/71.17 ± 12.1
XGBoost		92 ± 7.3/60.67 ± 15.53	
Random Forest		84.67 ± 11.39/55.5 ± 15.92	
SVM		78.5 ± 5.54/58.67 ± 14.84	
Logistic regression		76.67 ± 17.8/66.67 ± 19.58	
Elastic net		66 ± 13.05/73.17 ± 10.25	

ROC-AUC (%) on the <1 Year Test Set/1–2 Year Holdout Set.

**Table 3:** Comparative Model Performance for Ovarian Cancer Detection within 1 year before diagnosis.

Model Type	Sparsity	Node Feature	F1	Sensitivity	Specificity
GCN	None	TRUE	61.57 ± 3.5	81 ± 20.74	36.67 ± 32.06
	p = 0.2		53.95 ± 10.02	77 ± 22.8	20 ± 21.73
	p = 0.8		53.25 ± 12.28	70 ± 22.08	30 ± 29.81
	Min conn.		48.67 ± 18.07	64 ± 39.27	40 ± 41.83
	None	FALSE	65.04 ± 14.72	69 ± 24.08	66.67 ± 39.09
	p = 0.2		58.46 ± 14.43	60 ± 23.45	63.33 ± 36.13
	p = 0.8		66.4 ± 8.58	77 ± 17.89	56.67 ± 30.28
	Min conn.		52.19 ± 11.79	62 ± 26.83	46.67 ± 24.72
GATv2	None	TRUE	58.43 ± 13.32	56 ± 15.17	66.67 ± 42.49
	p = 0.2		42.44 ± 30.55	52 ± 46.04	60 ± 43.46
	p = 0.8		43.11 ± 26.84	48 ± 35.64	60 ± 43.46
	Min conn.		65.22 ± 8.51	96 ± 8.94	23.33
	None	FALSE	70.41 ± 18.16	79 ± 20.12	60 ± 41.83
	p = 0.2		61.33 ± 14.05	69 ± 28.37	63.33 ± 21.73
	p = 0.8		53.5 ± 30.85	80 ± 44.72	33.33 ± 47.14
	Min conn.		57.71 ± 11.77	73 ± 28.2	43.33 ± 40.14
XGBoost			66.55 ± 4.5	84 ± 16.73	50 ± 16.67
Random Forest			63.05 ± 8.14	96 ± 8.94	16.67 ± 23.57
SVM			61.19 ± 4.07	100 ± 0	3.33 ± 7.45
Logistic regression			69.97 ± 14.13	78 ± 17.89	63.33 ± 36.13
Elastic net			61.02 ± 13.79	78 ± 22.8	40 ± 34.56

**Table 4:** Comparative Model Performance for Ovarian Cancer Detection between 1 and 2 years before diagnosis.

Model Type	Sparsity	Node feature	F1	Sensitivity	Specificity
GCN	None	TRUE	55.55 ± 31.92	76 ± 43.36	40 ± 36.51
	p = 0.2		47.37 ± 27.6	71 ± 41.29	20 ± 21.73
	p = 0.8		56.32 ± 13.78	74 ± 21.62	33.33 ± 26.35
	Min conn.		51.6 ± 14.91	67 ± 32.71	36.67 ± 34.16
	None	FALSE	61.27 ± 10.57	73 ± 19.24	50 ± 31.18
	p = 0.2		63.56 ± 12.92	73 ± 13.04	56.67 ± 14.91
	p = 0.8		63.29 ± 11.18	85 ± 22.36	36.67 ± 24.72
	Min conn.		48.48 ± 15.56	55 ± 20	46.67 ± 27.39
GATv2	None	TRUE	33.71 ± 34	40 ± 46.9	70 ± 41.5
	p = 0.2		49.39 ± 30.35	58 ± 37.68	56.67 ± 34.56
	p = 0.8		48.5 ± 30.6	53 ± 40.56	60 ± 38.37
	Min conn.		67.51 ± 8.45	87 ± 18.57	46.67 ± 24.72
	None	FALSE	46.26 ± 20.74	58 ± 33.28	36.67 ± 21.73
	p = 0.2		45.56 ± 27.33	54 ± 35.78	46.67 ± 24.72
	p = 0.8		42.78 ± 30	65 ± 48.73	26.67 ± 34.56
	Min conn.		57.03 ± 32.75	72 ± 41.47	50 ± 23.57
XGBoost			46.46 ± 27.59	50 ± 31.42	70 ± 24.72
Random Forest			62.17 ± 8.57	96 ± 8.94	13.33 ± 21.73
SVM			61.19 ± 4.07	100 ± 0	3.33 ± 7.45
Logistic regression			39.05 ± 31.17	33 ± 26.36	80 ± 13.94
Elastic net			58.38 ± 19.77	59 ± 23.02	70 ± 13.94

## Data Availability

Raw assay data, except for CA125, are available upon request.
